# Predictors of aggression of university students

**DOI:** 10.4102/hsag.v25i0.1096

**Published:** 2020-02-20

**Authors:** Chris Myburgh, Marie Poggenpoel, Cornelius M. Fourie

**Affiliations:** 1 Department of Educational Psychology, Faculty of Education, University of Johannesburg, Johannesburg, South Africa; 2 Department of Nursing Science, Faculty of Health Science, University of Johannesburg, Johannesburg, South Africa

**Keywords:** aggression, descriptive, exploration, factor analyses, multivariate regression, predictors, university students

## Abstract

**Background:**

Post-secondary education forms the backbone of delivering high-powered persons in a country. Students are subjected to high levels of pressure to achieve success. This often promotes aggression towards self, others and even the environment. Predicting aggression is important as this can assist in managing aggression and the facilitation of the mental health of students. Little research has been published on the prediction of aggression in a university.

**Aim:**

To describe the prediction of aggression of students in one faculty in a university.

**Setting:**

Students in one faculty in a university.

**Methods:**

A deductive quantitative methodology was applied. Multivariate statistical techniques were used to answer the research questions. An online survey was conducted by using a questionnaire that comprised items related to biographic, personality and aggression aspects. Data were analysed by calculating Cronbach’s alpha values, various factor analyses (principal component analysis) and several multiple regression analyses to identify and describe the predictors of aggression.

**Results:**

Findings reflect that aggression can be predicted by intra- and interpersonal variables, such as ‘positive inclination towards others’, ‘positive inclination towards self’ and ‘acting responsibly towards self.’ Aggression is lower when a student’s positive inclination towards others is higher and towards self is lower and when a student acts with greater responsibility towards self.

**Conclusion:**

Students should understand and manage their own aggression. Overall, the findings showed that students are seemingly ‘adjusted’ conformists using an external locus of control. The facilitation of an internal locus of control and autonomous behaviour is imperative.

## Introduction, orientation and problem statement

Aggression is a phenomenon that is part of everyday human life and influences almost every level of an individual’s personal experience in a society by being varied, multi-faceted and diverse (Anderson & Huesmann 2003:296–323; Onukwufor 2013:62). Aggression often manifests from low-keyed, almost unintentional actions to intense, cold-blooded and well-planned actions towards those persons who are at the focus of the aggression (Anderson & Huesmann 2003:298–299; Mabitla 2009:8). Aggressive behaviour is a motivated behaviour and is often intended to cause injury, including physical injury to the self or others (Bandura 1973:2; Green 2001:3; Orton 1997:70; Sadock & Sadock 2007:149). These behaviours include verbal aggression, coercion, intimidation and destructive managerial styles that can have harmful psychological consequences for people. Aggressive behaviour often manifests as premeditated social ostracism of others. The impact of such behaviours should not be underestimated, nor the effects thereof on the self-esteem, social status and the happiness of the involved persons (Sadock & Sadock 2007:149–150).

The prevalence of aggression is challenging to demarcate. In this regard, Breet, Myburgh and Poggenpoel (2010:511–526) found that adolescent boys from lower socio-economic status environments tend to express more physical aggression compared to boys from higher socio-economic status environments who expressed more indirect and subtle aggression, yet still aimed at maximising destructive results. It is claimed by Anderson and Huesmann (2003:301) that persons with higher intellectual capabilities are more likely to exhibit subtle and indirect aggression compared to persons with a less-developed intellectual capability. The mosaic of aggression is thus diverse and complex.

Post-secondary education and training is the backbone of the development of future high-powered persons in any country. Students involved in university education are subjected to strenuous circumstances in their striving for success during training. This scenario often forms the breeding ground for aggression towards the self and others. Despite this, very little research has been published on the nature of the aggression experienced by university students. The researchers of this study, in accordance with the above fact, observed that aggression amongst university students is subtle and challenging. Furthermore, students are in a vulnerable situation. Lecturers are perceived as being in positions of authority. Lecturers are the gatekeepers to promotion and certification upon the successful completion of attended programmes. Furthermore, it can be expected that students might often compare and compete for the best achievements as this can bring about faculty and university prizes, such as monetary benefits and scholarships. This scenario can also have an impact on the already strenuous environment faced by a university student. Most students are preparing themselves for professions in which they will interact with other persons on a daily basis. The expectation is that aggression will be prominent because when human beings interact with each other there is a natural tendency for aggression to develop. The environment within which students are functioning often demands that they should be able to assess challenging situations that have the potential to become aggressive in nature.

Nobody willingly wants to be labelled as being aggressive. Therefore, it is expected that aggression at the university level will manifest in indirect and illusive ways. Students are expected to be alert when probed regarding their own experiences and perceptions of aggression. During interaction with other persons, it is essential to pre-empt when a situation has the potential to become aggressive. A number of prominent questions in this regard are the following: can one predict and pre-empt aggression of students as perceived by themselves? How does a person’s personality and psychological makeup, such as value systems and norm orientations, inclination towards responsibility, relationships with others, perceptions of oneself, dialogue and verbal interactions with others, play a role in the prediction of aggression?

## Aggression and predictors of aggression

Interaction with the self and others (Garbers 1972) entails the sensing, interpreting and comprehension of a person in his or her environment (Kneisl, Wilson & Trigoboff 2004:143; Sadock & Saddock 2007:281). Aggression usually manifests as negative name calling, the spreading of rumours, taking another person’s belongings, influencing others to dislike someone else and often destructive orientations such as being harsh towards oneself and others (Onukwufor 2013:64). Perceptions on aggression also refer to the perceptions of friends and peers concerning whether a specific person is aggressive.

Students and lecturers are in relationships with each other and norm representation and norm adherence in possible aggressive situations are at play. Toerien (2014: 65–75) found that lecturers experienced subtle and even explicit aggression from students. Thus, the presence of aggression on South African university campuses is a reality (Fourie 2017). Relationships with other persons and peers in the context of this study cannot be ignored while predicting students’ aggression. In this regard, having an aggressive inclination as is reflected in threatening others, an urge to hit or kick others, getting into fights and a peer’s perception of aggressiveness play a major role. However, within the context of peer relationships, the experience of safety with peers, their encouragement and communication with others, their norms and values should all be important.

Students are seemingly on guard regarding their own actions. They seemingly often explicitly use their intellect to manage their own behaviour and evade actions that could lead others to perceiving and labelling them as aggressive. When do students have a propensity for aggression? They appear more inclined towards implicit and indirect aggression. The values that a friend or peer adheres to can also play a role in an individual’s own orientations (Anderson & Huesmann 2003:306–308). Identifying with others, perceptions of other persons’ values and a reliance on own norms and values are fundamental in aggressive situations. Personal decision-making, exercising self-control, accepting responsibility and trusting one’s own norms are prominent when predictions of aggression are made.

In implementing structural equation modelling techniques, Bartlett and Anderson (2012:1870) found negative correlations between openness (*r* = -0.18), agreeableness (*r* = -0.47) and aggressive attitudes. Agreeableness (*r* = -0.31) and neuroticism (*r* = 0.50) correlated negatively and positively, respectively, for the same sample with aggressive emotions. Furthermore, aggressive attitudes correlated positively (*r* = 0.20) with violent behaviour. All coefficients were significant at the *p* < 0.001 level. In another investigation correlating personality, aggressive emotions and physical aggression using a structural equation model approach, Cavalcanti and Pimentel (2016:1–14) came to conclusions similar to those of Bartlett and Anderson (2012). In an extensive meta-analysis, Bettencourt et al. (2006:751) concluded that ‘some personality factors influenced aggressive behaviour under both neutral and provocation conditions …’. Although it seems as if little research refers to the factors being addressed in this article, it can be expected that apart from the research on the Big 5-personality traits, other personality factors can also be useful in assessing the prediction of aggression in a university.

There is a gap in the literature concerning the prediction of aggression of students at a university. Research was conducted on the Five-Factor Personality Traits model (extraversion, neuroticism, agreeableness, conscientiousness and openness to experiences and aggression) (Baumgardner & Crothers 2010), but not on the aspects mentioned above. Therefore, the research questions that directed this research were as follows: ‘are there personality dimensions that can be used to significantly predict and pre-empt perceptions of aggression of persons studying at a university?’ and ‘what are the implications for students to manage their own aggression?’

## Aim

The objective of this study was to describe the prediction of aggression of a group of students. In view of the findings, the implications for these students’ mental health are described.

## Research design and method

The study used a deductive quantitative methodology (Burns & Grove 2011:256). The design was contextual and descriptive in nature. Multivariate statistical techniques were used to answer the research questions. The data were analysed by calculating Cronbach’s alpha values, and various factor analyses (principal component analysis [PCA]). Multiple regression analyses were used to identify and describe the significance of predictors of aggression.

## Procedure

Ethical clearance was obtained from a registered ethics committee (ethical clearance number 2013–017, Faculty of Education, University of Johannesburg). Clearance was also given by the designated research official of the university who also managed the electronic collection of the data. Anonymous participation of students was voluntary, and the participants could benefit from completing the questionnaire as they had the opportunity to reflect on their own behaviour, their own experiences and the behaviour of other persons (Dhai & McQuoid-Mason 2011:14–15; Universal Declaration of Human Rights 2006). In this regard, the names of the university and the faculty are not included to ensure anonymity of the students invited to participate in the study.

A survey was conducted using a questionnaire that was distributed electronically via e-mail to all prospective participants in one faculty.

## Population and sample

A total of 3881 questionnaires were sent to students from which 266 usable questionnaires were electronically received back. (This meant that responses that answered at least 80% of all questions by the participants were included and questionnaires that did not meet this requirement were not included in the analyses). Students were invited to voluntarily participate in this investigation and no incentives were offered. The return rate is in line with the outcomes in other electronic investigations in related and other fields of research that report outcomes even as low as 9% (Hoonakker & Carayon 2009; Martinez & Kalliny 2012; Fritz & Silva 2018). According to these authors, there is often an unwillingness to participate in electronic surveys. The outcome of this specific investigation is acceptable especially in view of the nature of the selected population; the approach followed was that this research was exploratory in nature and that we did not intend to make generalisations but rather explored trends amongst students in this faculty of the university. Eventually, a total of 227 respondents’ questionnaires remained after the data were cleaned. Thecleaning process of the questionnaires was managed by a designated university official to ensure the anonymity of participating students. The 227 questionnaires formed the data for this investigation.

## Questionnaire

The instrument was a questionnaire consisting of biographic items, items on aggression and potential predictor items of aggression. The questionnaire (available on request) is the culmination of a literature study and the results from numerous research projects. These comprise both qualitative and quantitative studies.

The items included aggression and predictors of aggression such as self-perception, values and norms, responsibility and relations with other persons (85 questions in total). Each question item was assessed on a 5-point Likert-type scale (Burns & Grove 2011:357–358) ranging from 1 ‘extremely uncharacteristic of me’ to 5 ‘extremely characteristic of me’. Individual ordinal items were grouped into factors based on exploratory factor and internal consistency analysis. Interval variables were derived per factor by averaging the individual items making up the factors.

## Research results and contextualisation

In the following sections, validity and reliability are discussed along with the results of the various multiple regression analyses. It should be emphasised that this is an exploratory research and that none of these factors are the results of previous research.

### Validity and reliability

Validity and reliability (Burns & Grove 2011:332–335; Walker 2010:52) were assessed in the following manner: items in the questionnaire relevant to aggression and the predictor factors of aggression were identified. Various PCAs were conducted (IBM Statistical Package for the Social Sciences [SPSS] Statistics 25 package 2018). Items with factor loadings less than 0.5 and loading simultaneously on two factors were removed. The Kaiser–Meyer–Olkin (KMO) measure of sampling adequacy of the factor analyses (see Tables 1–4) ranged from 0.622 for value systems assessed as ‘mediocre’ to 0.837 assessed as ‘great’ (Field 2005:649–650). Furthermore, Bartlett’s coefficients to assess sphericity were significant (*p* < 0.001) in all cases (Field 2005:652); this means that the KMO values are all above 0.5 and the Bartlett’s test is significant in all cases. The identified factors were used for further investigation of aggression (Field 2005). Finally, the explained variance of the various factor analyses ranged from 48.48% to 62.21%. The reliability of each factor was assessed using the calculated Cronbach’s alpha value.

### Ethical consideration

The National Health Research Ethics Council (NHREC) registration number was REC-110613-036. Ethical approval to conduct the study was obtained from the Faculty of Education, University of Johannesburg Ethics Committee (clearance number: 2017-055).

### Results and discussion of results

Some of the demographics of the sample (consisting of 227 participants) include the fact that 162 participants were women, 85 were post-graduate students and the sample had a mean age of 27.87 years.

Four different factors describing aggression were identified (Table 1). Factor 1d was identified as *verbal aggression* (mean = 1.56, standard deviation [SD] = 0.63), as it mainly refers to acts of verbal aggression towards other persons. Factor 2d was identified as *aggressive inclination towards others* (mean = 1.78, SD = 0.78). Factor 3d was *aggression towards self* (mean = 2.43, SD = 1.07), as it refers to being ‘harsh’ towards oneself. Factor 4d was identified as *aggression towards others* (mean = 2.51, SD = 0.89), as it describes being almost open, direct and hostile towards other persons.

**TABLE 1:** Item loadings with respect to three factors describing aggression and Cronbach’s alpha reliability coefficients: Rotated component matrix.†, ‡, §, ¶

Items	Factors			
	1	2	3	4
B57: I sometimes tend to say bad things about people behind their backs	0.793	**-**	**-**	**-**
B58: I sometimes call people by negative names	0.753	**-**	**-**	**-**
B60: I sometimes tell peoples’ secrets to other people	0.679	**-**	**-**	**-**
B65: I sometimes try to influence people to dislike a specific person with whom I am angry	0.638	**-**	**-**	**-**
B53: I sometimes tell false stories about people	0.596	**-**	**-**	**-**
B73: I get into fights a little more than an average person does	**-**	0.724	**-**	**-**
B45: Given enough provocation, I may hit another person	**-**	0.675	**-**	**-**
B49: I sometimes tend to kick other people when I am upset	**-**	0.666	**-**	**-**
B74: I have threatened people I know	**-**	0.610	**-**	**-**
B79: I sometimes see myself as being harsh towards myself	**-**	**-**	0.817	**-**
B77: I sometimes view myself as aggressive towards myself	**-**	**-**	0.787	**-**
B59: I sometimes tend to take things from other people without their permission	**-**	**-**	0.621	**-**
B62: I sometimes write small notes criticising other people	**-**	**-**	0.518	**-**
B68: My friends say that I am somewhat aggressive towards other persons	**-**	**-**	**-**	0.809
B82: Some of my friends think I am a hothead	**-**	**-**	**-**	0.696
B85: I often find myself disagreeing with people	**-**	**-**	**-**	0.681
B64: I sometimes criticise peoples’ appearance (i.e. their hair styles, clothes, etc.)	**-**	**-**	**-**	0.505

Extraction method: Principal component analysis. Rotation method: Varimax with Kaiser normalisation, explained variance is 58.26%.

† , Rotation converged in seven iterations.

‡ , Kaiser–Meyer–Olkin measure of sampling adequacy = 0.837.

§ , Bartlett’s test of sphericity *p* = 0.000.

¶ , Cronbach’s alpha: 0.854 (17 items) – factor 1d: 0.800 (five items); factor 2d: 0.704 (four items); factor 3d: 0.758 (four items); factor 4d: 0.696 (four items).

Factor 5i was identified as *positive inclination towards others* (mean = 4.08, SD = 0.70), as the items on this factor indicated an openness and positive perception towards other persons. Factor 6 was identified as *positive inclination towards self* (mean = 2.79, SD = 1.02), as it refers to being positive towards oneself (Table 2).

**TABLE 2:** Item loadings with respect to two factors describing the self-perception of the participants and Cronbach’s alpha reliability coefficients: Rotated component matrix.†, ‡, §, ¶

Items	Factors	
	5	6
B72: I am always seen as being friendly towards others	0.814	-
B71: I am always approachable by other persons	0.778	-
B75: I view myself as usually being supportive towards other persons	0.666	-
B66: I view myself as sociable towards others	0.639	-
B80: I view myself as being understanding towards myself	-	0.826
B81: I view myself as loving myself	-	0.814
B84: I view myself as caring towards myself	-	0.772

Extraction method: Principal component analysis.

† , Rotation method: Varimax with Kaiser normalisation, variance explained is 60.23%.

‡ , Rotation converged in three iterations.

§ , Kaiser–Meyer–Olkin measure of sampling adequacy = 0.668.

¶ , Bartlett’s test of sphericity *p* = 0.000.

†† , Cronbach’s alpha: 0.780 (seven items) – factor 5i: 0.684 (four items); factor 6i: 0.740 (three items).

Factor 7i was identified as *acceptance of important persons’ values* (mean = 3.31, SD = 0.90), as it refers to the acceptance of important personal values. Factor 8 was identified as *acceptance of peers’ values* (mean = 3.06, SD = 0.86) (Table 3).

**TABLE 3:** Item loadings with respect to two factors describing the value system of the participants and Cronbach’s alpha reliability coefficients: Rotated component matrix. †, ‡, §, ¶

Items	Factors	
	7	8
B2: I usually accept my peers’ beliefs about what are important in life		0.841
B3: I usually accept that my peers will do what they say	-	0.733
B1: I usually live according to the religious values of my peers	-	0.591
B16: I usually accept important persons in my life as being good examples for me on sexual issues	0.871	-
B17: I usually accept important persons in my life as being good examples for me on relationships	0.836	-
B15: I usually accept that important persons in my life will do what they say that they will do	0.662	-

Extraction method: Principal component analysis.

† , Rotation method: Varimax with Kaiser normalisation, explained variance is 62.21%.

‡ , Rotation converged in three iterations.

§ , Kaiser–Meyer–Olkin measure of sampling adequacy = 0.624.

¶ , Bartlett’s test of sphericity *p* = 0.000.

†† , Cronbach’s alpha: 0.858 (six items) – factor 7i: 0.715 (three items); factor 8i: 0.558 (three items).

Factor 9i describes participants’ *acting responsibly towards self* (mean = 3.90, SD = 0.63), as it indicates doing what is correct, acting with self-control in tense situations, accepting responsibility, not surrendering own ideas and not willing to negate own ideas. Factor 10d describes participants’ *reliance on own norms* (mean = 3.39, SD = 0.91), as it indicates a willingness to judge peers’ behaviour and a willingness to differ from peers (Table 4).

**TABLE 4:** Item loadings with respect to two factors describing the Norm System of the participants and Cronbach’s alpha reliability coefficients: Rotated component matrix †, ‡, §, ¶

Items	Factor	
	9	10
B8: I differ from my peers on religion-related issues	**-**	0.801
B9: I differ from my peers on study-related issues	**-**	0.788
B7: I differ from my peers on sexuality-related issues	**-**	0.732
B42: I accept responsibility for the things that I do	0.746	**-**
B41: I always exercise self-control in tense situations	0.642	**-**
B88: I negate my own ideas in favour of other persons when I am pressured (Transpose)	0.621	**-**
B35: I always do what I think is correct (regardless of what others might say)	0.558	**-**
B87: I surrender my ideas or will to others (transpose)	0.555	**-**

Extraction method: Principal component analysis.

† , Rotation method: Varimax with Kaiser normalisation, explained variance is 48.48%.

‡ , Rotation converged in three iterations.

§ , Kaiser–Meyer–Olkin measure of sampling adequacy = 0.622.

¶ , Bartlett’s test of sphericity *p* = 0.000.

†† , Cronbach’s alpha: 0.779 (eight items) – factor 9i: 0.598 (five items); factor 10i: 0.691 (three items).

In Table 5, the significance of correlations between the 10 factors is given, indicating the relationships that are analysed in greater detail in the exploratory analysis.

**TABLE 5:** Significance of two-sided correlations between factors (*N* = 227).

Factors	1d	2d	3d	4d	5d	d	7d	8d	9d	10d
1d	1	**-**	**-**	**-**	**-**	**-**	**-**	**-**	**-**	**-**
2d	0.478**	1	**-**	**-**	**-**	**-**	**-**	**-**	**-**	**-**
3d	0.414**	0.429**	1	**-**	**-**	**-**	**-**	**-**	**-**	**-**
4d	0.405**	0.339**	0.446**	1	**-**	**-**	**-**	**-**	**-**	**-**
5d	−0.255**	−0.231**	−0.208**	−0.148*	1	**-**	**-**	**-**	**-**	**-**
6d	0.346**	0.364**	0.674**	0.482**	−0.108	1	**-**	**-**	**-**	**-**
7d	−0.011	−0.030	−0.028	0.015	0.189**	0.030	1	**-**	**-**	**-**
8d	−0.037	−0.081	0.006	0.026	0.148*	−0.034	0.218**	1	**-**	**-**
9d	−0.398**	−0.350**	−0.250**	−0.173**	0.220**	−0.329**	0.064	0.024	1	**-**
10d	0.033	0.010	0.104	0.075	−0.016	0.086	0.026	−0.116	−0.019	**-**

* , Significance at the 5% level.

** , Significance at the 1% level.

Factor 1d: verbal aggression; factor 2d: aggressive inclination towards others; factor 3d: aggression towards self; factor 4d: aggression towards others; factor 5i: positive inclination towards others; factor 6i: positive inclination towards self; factor 7i: acceptance of important persons’ values; factor 8i: acceptance of peers’ values; factor 9i: acting responsibly towards self; and factor 10i: reliance on own norms.

## Exploratory inferential analyses of the predictors of aggression

The six predictor factors of aggression (see Tables 2–4; factors 5i–10i [‘i’ indicates an independent variable]) were used as independent variables in further analyses. Aggression (total of 17 items, see Table 1 [mean = 2.04, SD = 0.63]) and the four aggression factors (factors 1d–4d [‘d’ indicates a dependent variable]) were used as dependent variables.

Firstly, and before the multiple regression analyses were conducted, the significance of differences between genders and undergraduate versus post-graduate groups was assessed. No substantial and significant differences in factors were identified between the compared groups (not reported in this article). In general, Anderson and Huesman (2003:299–301) indicated that in society there are indications that age and gender play a definite role in the manifestation of aggression, which is different from what was found in this study amongst subgroups of the sample group of students.

Secondly, 15 multiple regression analyses were conducted by using various independent variables used to predict aggression. Five variables describing aggression were used in the analyses, namely, aggression total and the four factors on aggression: (1) verbal aggression (factor 1d), aggressive inclination towards others (factor 2d), aggression towards self (factor 3d) and aggression towards others (factor 4d). The six predictor variables (factors) that were used in the multiple regression analyses were the following: positive inclination towards others (factor 5i), positive inclination towards self (factor 6i), acceptance of important persons’ values (factor 7i), acceptance of peers’ values (factor 8i), acting responsibly towards self (factor 9i) and reliance on own norms (factor 10i).

In Table 6, the significance of a specific predictor on the particular factor of aggression is presented on either the 1% (**) or 5% (*) level of significance. Per aggression factor, the following multiple regressions were executed: for the total group (*n* = 227), for the female subsample (*n* = 162) and for the male subsample (*n* = 65). The significances of predictor variables (factors 5i–10i [6 in all]) are given in Table 6. Thus, each line (row) in Table 6 represents the significance of a specific multiple regression equation. Furthermore, if a factor was not identified as a significant predictor in at least two of the regression analyses (i.e. for the total group, the females and/or the males), that factor was assessed as not being a ‘strong’ predictor of the aggression total and the four factors of aggression.

**TABLE 6:** Significance on the 1% or 5% level of significance of a specific factor as a predictor of aggression: Linear multiple regression.

Factor	5i	6i	7i	8i	9i	10i	Adj *R*^2^	*F*	*F* (sig)
**Aggression**									
All	**(-)	**	-	-	**(-)	-	0.463	33.52	0.000
Females	**(-)	**	-	-	-	-	0.445	22.53	0.000
Males	-	**	-	-	**(-)	-	0.530	13.03	0.000
**Verbal aggression (factor 1d)**									
All	**(-)	**	-	-	**(-)	-	0.217	11.46	0.000
Females	*(-)	**	-	-	*(-)	-	0.165	6.29	0.000
Males	*(-)	-	-	-	**(-)	-	0.382	7.59	0.000
**Aggressive inclination towards others (factor 2d)**									
All	*(-)	**	*	-	**(-)	-	0.195	10.14	0.000
Females	*(-)	**	-	-	**(-)	-	0.150	5.72	0.000
Males	-	**	-	-	*(-)	-	0.309	5.77	0.000
**Aggression towards self (factor 3d)**									
All	**(-)	**	-	-	-	-	0.465	33.70	0.000
Females	**(-)	**	-	-	-	-	0.449	22.84	0.000
Males	-	**	-	-	-	-	0.507	11.99	0.000
**Aggression towards others (factor 4d)**									
All	-	-	**	-	-	-	0.226	12.00	0.000
Females	-	-	**	-	-	-	0.216	8.41	0.000
Males	-	-	**	-	-	-	0.211	3.85	0.003

* , Significance at the 5% level of significance as a predictor.

** , Significance at the 1% level of significance as a predictor; A ‘-’ next to either * or ** indicates that the significant predictor is predicting negatively towards aggression.

Dependent factors: aggression total; 1d: verbal aggression; factor 2d: aggressive inclination towards others; factor3d: aggression towards self; and factor 4d: aggression towards others.

Predictor factors: 5i: positive inclination towards others; 6i: positive inclination towards self; 7i: acceptance of important persons’ values; 8i: acceptance of peers’ values; 9i: acting responsibly towards self; and 10i: reliance on own norms.

From Table 6 it follows that overall the findings are:

- Aggression total (17 items) is significantly predicted by a positive inclination towards others (negative correlation) (5i); a positive inclination towards self (positive correlation) (6i) and acting responsibly towards self (negative correlation) (9i). The finding that a positive inclination towards self positively predicts aggression in itself is challenging to explain; nevertheless, it cannot be ignored. Research has found that individuals with a high self-esteem are more prone to anger and highly aggressive when their self-image is threatened (Baumeister, Smart & Boden 1996; Kernis, Brockner & Frankel 1989). This seemingly contentious finding can be explained by the fact that when the perception of a person coming across as fairly positive towards themselves is challenged on aspects dearly valued, often uncertainty can cause them to become aggressive. Noting that the level of aggression reported has a relatively low base (i.e. ratings for different forms of aggression are relatively low) and there is a strong correlation between positive inclination towards self and aggression towards self, it might be possible that students with high positive inclination are also likely to be highly self-critical, and hence the positive correlation. Another possible explanation is provided further. The previous observation that a large proportion of these students could be first-generation students can contribute towards supporting this explanation.
- Secondly, verbal aggression (factor 1d) is predicted by factors 5i, 6i and 9i, namely, a positive inclination towards others (negative correlation) (5); a positive inclination towards self (positive correlation, see explanation offered under the first bullet point above) (6); and acting responsibly towards self (negative correlation) (9).
- Aggressive inclination towards others (factor 2d) is predicted by a positive inclination towards others (negative correlation) (5i); positive inclination towards self (positive correlation, see the first bullet point above) (6i); and acting responsibly towards self (negative correlation) (9i).
- Aggression towards the self (Factor 3d) is predicted by a positive inclination towards others (negative correlation) (5i); and a positive inclination towards self (positive correlation, see the first bullet above) (6i).
- Aggression towards others (Factor 4d) is predicted by acceptance of important persons’ values (7i).
- Overall acceptance of peers’ values (8i) and reliance on own norms (10i) did not contribute towards significantly predicting any of the aggression factors.

The mosaic and intricacies revealed by the findings shown above will be interpreted and contextualised against the thoughts of Riesman posited in his classic groundbreaking work *The Lonely Crowd* (Riesman, Glazer & Denney 1961). In this work, Riesman describes three types of ‘adjusted’ conformists, namely, tradition-directed, inner-directed and other-directed persons (Riesman et al. 1961; The Big Answer 2019). According to The Big Answer (2019; see also Riesman et al. 1961), tradition-directed persons mostly relate to immigrants from peasant societies, such as labourers and farmers; inner-directed persons are those who are guided and controlled by their own superegos and as such are guided by guilt that enforces them to act according to their upbringing; and finally other-directed persons that are also adjusted conformists. The important comments are that inner-directed persons are guided and controlled by their ‘superego forces’ that were instilled by parents. This controls their decisions (Riesman et al. 1961; The Big Answer 2019). Furthermore, other-directed persons are controlled by ‘that’s what my peers expect of me’ and tradition-directed persons are controlled by ‘that’s what we’ve always done’ (The Big Answer 2019; see also Riesman et al. 1961). Extrinsic pressures therefore play a role in decision-making and perceptions in all three groups of ‘adjusted’ persons and clearly indicate an external locus of control and absence of an inner locus of control (Piraino 2013).

From the findings above, and interpreted against Riesman’s ideas, it follows that aggression (total) is lower when a student’s positive inclination towards other persons is higher (5), when a student’s positive inclination towards self is lower (6) and when a student acts with higher responsibility towards self (9). The content of factors 6 and 9 can be indicative thereof that students can make a distinction between being overly self-assertive and being self-centred. Students’ aggression is fuelled when others are less focussed upon; high emphasis on self is prominent and low responsibility towards self is prevalent. Furthermore, when a person focusses on others and is responsible towards self (behaves sensibly) and is less inclined to self (not in love with themselves), he or she might tend to be less aggressive. This can be indicative of an inner-directed person (Riesman) and an absence of autonomous decision-making, as the focus of aggressive behaviour is an emphasis on the self as opposed to others, but also having a low responsibility towards self. This picture is challenging, as, on the one hand, these students’ aggression is low when they perceive having a higher positive inclination towards others and act responsibly towards themselves, as opposed, on the other hand, to perceiving themselves as having a less positive inclination towards themselves. The implications of this finding are unclear and necessitate further research. Verbal aggression (1d) and aggressive inclination towards others (2d) are predicted by the same factors as aggression total. The same underlying motivations of students according to their perceptions could be at play as discussed above.

The prediction of aggression towards self (3d) is lower when a positive inclination towards others is higher (5i), and it is lower when a positive inclination towards self is lower (9i). It thus seems as if the presence of an external locus of control is present. This can be indicative of other-directedness in decision-making and aggressive actions. Their aggression is lower when they have a high acceptance of others and low inclination towards themselves. Again, their aggressive inclination is defined by an external rather than an internal locus of control. Although not clearly crystallised, it seems as if this finding might have implications for identity formation and crystallisation versus the role of relationships with other persons. For instance, much depends on what is meant by ‘positive inclination towards self’, which is measured by ‘loving self’ and ‘caring for self’ in this article. If this is too high, it might be unhealthy, for example, a representation of narcissistic behaviour. It could be that less self-absorbed persons are less aggressive than those who are very self-absorbed (positively inclined towards self). There is a negative correlation between responsibility towards self and positive inclination towards self, suggesting that when persons are self-absorbed they are less likely to report responsible behaviour. So the result makes sense if a high rating for ‘positive inclination towards self’ is not always assumed to be a healthy state (i.e. a high rating for positive inclination towards self might not be a healthy state).

Furthermore, aggression towards others (4d) is predicted only by acceptance of important persons’ values (7i). The lower the acceptance of important persons’ values, the lower the aggression. This is an important finding that could indicate that the more these students are not accepting important persons’ values, the lower their aggression will be, and they are possibly less inclined towards tradition-directed decision-making. This could be a sign that they are in a process of becoming certain of their own identities and that this certainty makes them less prone to accept important persons’ values, and thus less prone to act aggressively. This has definite implications for identity certainty of students. Should students be certain of who they are and be able to focus on their own values rather than on important other persons’ values, the less aggressively they might act. Overall, it nevertheless gives an indication that these students could be ‘adjusted’ conformists as there is a link between aggression and being tradition-oriented.

These findings, to a certain extent, might identify challenges in the self-perceptions of these students when reflecting on aspects of intra- and interpersonal relationships and aspects of aggression. Although not explicit in the findings, it might be an indication that these students are not able to clearly demarcate the relationship between themselves and others. Although the above findings are exploratory in nature, there are definite indications that intra- and interpersonal relationships can be used to facilitate the mental health of students at a university. These findings show that students’ perceptions reflect a relationship between intra- and interpersonal relationships and aggression.

## Implications

Some implications of the above findings are that this group of students in one way or the other are affected by being ‘adjusted’ conformists (Riesman) concerning their perceptions of aggression or when decisions are made concerning aggression. This is regardless of whether they are tradition-directed, inner-directed and other-directed persons. The implication of this finding is that aggression in its variety of presentations is acted upon by ignoring autonomous behaviour by using an external locus of control. The mental health challenges in this regard are demanding. Nevertheless, to unpack this implication it is necessary to contextualise the issues involved. From these findings it follows that when perceptions of other persons are positive, and when there exists a less positive perception of self (as represented by being less self-absorbed or even less narcissistic) together with high responsibility towards the self, it might predict lower aggression. This can play a role in the perceptions and management of aggression, whether it is aggression (total), verbal aggression or an aggressive inclination towards other persons. Although the findings might be clear, the implications thereof are complex. The interplay between the self and others and responsibility towards self needs unpacking through further research. If these observations are precise, then the relationship between intra- and interpersonal relationships amongst most of these students needs intervention as their mental health might be somewhat at risk. For optimal mental health a healthy balance between self and others within a specific social environment is needed. Healthy self-love seemingly needs to be facilitated in those students who demonstrate a high level of aggression (Myburgh, Poggenpoel & Tolsma-Hastings 2017).

Although this is part of an intensive research project spanning more than two decades and consisting of more than 100 associated published projects of qualitative and quantitative nature, this is the first project focussing on the prediction of relationships between intra- and interpersonal relationships and aggression. This is important as interactions between human beings are inherently power relationships (Foucault 1982). It is refreshing that although no such similar projects could be identified in the literature, there are indeed relationships that can assist in predicting aggression of students in order to facilitate their mental health. Furthermore, it is also important that these relationships are highly specific, namely, that aggression can indeed be predicted (Kohn 1988; The Valley Behavioural Health System 2015). An aggressive inclination of a person is an internal process, attitude and ‘motivating force’. When this finding is linked with the observation that all behaviours are motivated (Ching 2015; Ryan 2012), it becomes clear that the university, through its counselling services, has an obligation to assist students to identify, address and stimulate a decrease of such an inclination. An individual’s reflection on his or her own thoughts and behaviours concerning becoming aggressive might assist in counteracting such an inclination. Universities are places where stress often reaches high levels because of challenges that students and lecturers are confronted with.

If this exploratory research correctly reflects interrelationships between intra- and interpersonal relationships, then this could be used to facilitate the mental health of students by assisting them to distinguish between intra- and interpersonal relationships and focus on being assertive without transgressing towards other persons. The nurturing of healthy self-love (Myburgh et al. 2017) seems to be important in this case.

The participants in this research were either already in a profession or were preparing to enter a profession. Therefore, support and facilitation to address challenging mentally unhealthy behavioural patterns should be addressed. Participants in this research were seemingly not unaware of these challenges, as this was excellently demonstrated by their responses to the various question items. All interventions should be directed at the facilitation of addressing an implicit awareness of possible destructive attitudes and behavioural motivations in interacting with oneself and others. Thus, the participants should be assisted to revert to a healthy addressing of intra- and interpersonal relationships in demanding and everyday ordinary challenging situations. The implications that were briefly addressed are viewed as imperative to facilitate mentally healthy relationships (intra- as well as interpersonally), taking cognisance of the important fact that power relationships need not be based on autocratic approaches towards one another, or even one’s self. From the results of this project it follows that these students have, to a certain extent, an external locus of control. It will be necessary to help them in developing an internal locus of control to support them in acting autonomously when being confronted with situations that can develop into aggressive encounters.

Finally, certain variables included in this project did not predict aggression or various aspects of aggression. These include acceptance of peer’s values (8) and reliance on own norms (10). The implications of these findings can be that either peers do not play a role or that the students are already sure of their own identity. Both derivations can be challenged, making the clarification of the findings even more challenging. If this is true, then the question can be asked why reliance did not on its own norms contribute towards predicting aggression. Further research to unpack these aspects is imperative. The clarification of one’s own norm orientation and support to adhere to own norms seems to play a definite role in the management of aggression, and this finding can be used to facilitate an internal locus of control. Cascio (2016) states that when individuals reflect on their values, resilience is often fostered and aggression decreases. Although adherence to norms plays a significant role only in the prediction of a decrease of active aggressiveness, norms (either silently or explicitly) always undergird aggressive situations. Norms are the foundations upon which situations in practice and at universities should be functioning and, as such, they are fundamental in the facilitation of the mental health of involved individuals. Responsibility and honouring responsibility in all situations counteract active aggression and can reflect having an internal locus of control. Nurturing a constructive social climate (Myburgh et al. 2017), and positive interpersonal relationships to facilitate responsibility towards self and others, is imperative (Li et al. 2014; Locasia 2003; St-Pierre 2011; Weiner 1995). Addressing active aggression should thus also emphasise the responsibility of individual students during the development of active aggression.

A positive inclination towards self, self-nurturing and a healthy self-love seems to be imperative in aggressive situations. In university situations, cognitive distance between individuals is the order of the day. This finding stresses the imperative and inherent role that affect plays. A student is not only an intellectual being but also a spiritual being encompassing body, mind and spirit. Emotions, affections and a positive inclination towards self can be used to counteract aggression towards oneself (Pietersma & Dijkstra 2012; Cascio 2016; Thomaes et al. 2009).

A whole person approach is needed, where the integration of body, mind and spirit is aimed at facilitating an internal locus of control and the mental health of individuals and groups overall in an often clinical and cold university atmosphere. Departments, faculties and the university should aim to become caring facilities. When lecturing and administrative staff really become caring towards the students they serve, only then aggression in universities might be lowered and universities may become places where aggression could be managed in a healthy manner.

## Limitations

It is important to take cognisance of the fact that the self-perceptions of these students were used; however, persons are living and interpreting the world, their interrelationships and their intrapersonal relationships through their own perceptions. Furthermore, no cultural interpretations or variations are reflected in this research, although this may play a role in persons’ acting and interpreting of aggression and variations thereof. Another aspect that should be taken into consideration is that the data are obtained from a cross-sectional sample and that claims concerning causality should be viewed against this background. It is acknowledged that treating Likert-scaled data as interval data can be questioned. Finally, one of the biggest challenges is that data from electronic surveys are often not a precise reflection of the population they were originating from. In the case of this investigation, the findings are only applicable to this specific sample and can merely be used as indicators of certain trends.

## Conclusion

Aggression is part and parcel of this university’s everyday functioning and cannot be ignored. In this article, the findings indicate that aggression can be predicted by the norm orientation, taking responsibility and an aggressive inclination towards self and others. It followed from the analysis of trends in the data that these students are to a certain extent ‘adjusted’ conformists using an external locus of control to manage aggression. The facilitation of an internal locus of control and autonomous acting of individuals (students) are imperative for the promotion of their mental health.
